# One-Step Synthesis of Mesoporous Chlorine-Doped Carbonated Cobalt Hydroxide Nanowires for High-Performance Supercapacitors Electrode

**DOI:** 10.1186/s11671-018-2791-z

**Published:** 2018-12-27

**Authors:** Yang Zhao, Shaobo Liu, Baihui Zhang, Jianfei Zhou, Wenke Xie, Hongjian Li

**Affiliations:** School of Physics and Electronics, Central South University, Changsha, 410083 China

**Keywords:** Supercapacitor, Nickel foam, One-step method, High performance, Porous nanomaterials

## Abstract

Self-stabilized and well-defined chlorine-doped carbonated cobalt hydroxide nanowires have been obtained as a binder-free electrode via a facile method. The Co material has a unique well-defined needle-like structure, composed of highly aligned monomer with the diameter of about 3–10 nm and numerous surface pores, which makes it have potential for high-performance electrochemical capacitors. The test results show the directly acquired Co-ClNWs(NiE) electrode in three-electrode system can reach the specific capacity of more than 2150 F/g under the current density of 1 A/g, accompanied by a good cycling stability of 94.3% capacitance retention after 500 cycles, and exhibits a high energy density of 41.8 W h/kg at the power density of 1280.7 W/kg when using it as the positive electrode of an asymmetric supercapacitor. After making a comparison of the current material with the conventional electrodes, we can find that a better electrochemical performance can be achieved with a more convenient one-step method. Therefore, we, in this work, may provide a new type of manufacturing concept for future electrode treatment.

## Introduction

As a kind of energy storage and conversion device, supercapacitor has attracted tremendous attention owing to its fast charging and discharging rate, high power density, long cycle life, and high reliability advantages [1, 2]. In recent years, it has supplemented the deficiency of the traditional energy storage and conversion equipments in many important applications and prospect fields such as military electronic equipment, electric vehicles, portable computers, etc. [3–7]. Generally, supercapacitors can be divided into two types according to their different electron storage mechanisms: traditional electrical double-layered capacitors (EDLCs) which store energy by accumulation of charges in the electrical double layer via electrostatic interactions, and pseudocapacitors which store energy via Faradaic redox reaction at electrode surface [8–11]. Among the various pseudocapacitance materials, ruthenium oxide exhibits excellent electrochemical performance, but high cost, low porosity, and toxic nature severely limit its commercial application [12]. Therefore, some cheaper and more environmentally friendly but highly capacitive metal oxides/hydroxides such as NiO, Co_3_O_4_, Fe_3_O_4_, Fe_2_O_3_, V_2_O_5_, Co(OH)_2_, and Ni(OH)_2_ have become the most promising candidates [13]. Co(OH)_2_, displaying the obvious advantages of well-defined reversible redox reactions with high theoretical specific capacity, has been considered as a particularly attractive potential material [14]. The study finds that high capacitance performance reflects in a special morphological structure with high specific surface area [6, 15–18]. From previous reports, Mahmood and his co-workers synthesized chlorine-doped carbonated cobalt hydroxide (Co(CO_3_)_0.35_Cl_0.20_(OH)_1.10_1.74H_2_O) nanowires with extraordinary capacitance and excellent energy density along with high rate capability and stability. Such high capacitance and energy densities are thought to be attributed to the unique structure of the Co(CO_3_)_0.35_Cl_0.20_(OH)_1.10_ nanowires, in which hydrophilic nature can significantly enhance the wettability of the electrode surface, and the existence of counter structure stabilizer anions (Cl^−^ or/ and CO3^2−^) effectively controls the polarization of the electrode [19]. Inspired by the superiorities of such work, the prospect in terms of optimizing the structural and electrochemical properties by doping of some elements into Co(OH)_2_ is foreseen.

At the same time, in order to obtain high specific surface area and other special morphology, the researchers begin to innovate in structure [17, 20–23]. When active material was attached to the other electrode material surface, it could form a parcel core-shell structure or layered three-dimensional structure, which could ensure the effect of the active material and electrolyte ion contact in improving the reaction efficiency. For example, Shude Liu and his co-workers proposed a supercapacitor electrode comprising a three-dimensional self-supported hierarchical MnCo-layered double hydroxides@Ni(OH)_2_ [MnCo-LDH@Ni(OH)_2_] core–shell hetero structure on conductive nickel foam [24]. The resultant MnCo-LDH@Ni(OH)_2_ structure exhibited a high specific capacitance of 2320 F/g at a current density of 3 A/g, and a capacitance of 1308 F/g was maintained at a high current density of 30 A/g with a superior long cycle lifetime. However, due to the different characteristics of materials, the preparation method has been facing with the problems of complicated operation, harsh reaction conditions, and low success rate. Therefore, a more handy preparation measure to obtain uniform and orderly electrode materials with high electrochemical performances is highly desired [25].

In this paper, the mesoporous chlorine-doped carbonated cobalt hydroxide nanowires (Co-ClNWs) are directly grown on the nickel foam to prepare the nickel foam electrode (Co-ClNWs(NiE)) by a facile one-step hydrothermal method based on the performance advantages of Co(OH)_2_. The electrochemical performance test is performed with Co-ClNWs(NiE) directly as the working electrode, which provides a key measure to enhance both specific capacitance and energy density for its reasonable realization of the inner active sites of the bulk materials for storage energy. Meanwhile, the performance comparison is performed with the conventional electrode. It provides a feasible reference method for the increasement of capacitance and the application development of Co materials, and also offers new ideas for the structure and production of capacitor industrialization in the future.

## Methods

### Synthesis of Co-ClNWs on Ni Foam

Ni foam was obtained from Canrd Co., Ltd., China. Prior to use, it was treated with 0.5 M HCl under ultrasonic for 0.5 h, and then dried at 80 °C for 12 h after washing by a great amounts of deionized water and ethanol to remove surface ions. All other chemicals were analytical grade and purchased from Sinopharm Chemical Reagent Co., Ltd. in China without further treatment before use.

Firstly, 3.5 g CoCl_2_·6H_2_O and 0.9 g CO(NH_2_)_2_ were dissolved in 100 mL deionized water under magnetic stirring, lasting for 30 min until the solid was completely dispersed and dissolved. The obtained homogeneous solution was then transferred into stainless steel autoclave with several clean nickel foams fixed by stainless steel clips (the quality of the nickel foam was measured in advance), making sure the nickel foams are completely submerged, and placing at 120 °C with a thermal reaction of 20 h. After cooling to room temperature, the nickel foams were fetched out and washed with deionized water to remove impurities adhered on the surface. Finally, the samples were selected in the vacuum, drying in an oven for 10  h for use.

### Material Characterizations

The structures and morphologies of the products were analyzed by field-emission scanning electron microscopy (SEM MIRA3 TESCA) and transmission electron microscope (TEM FEI Tecnai). X-ray diffraction (XRD) patterns were collected with a SIEMENSD500 diffractometer with Cu Kα radiation (λ = 0.15056 nm). X-ray photoelectron spectroscopy (XPS) was carried out on ESCALAB 250 with Al Kα radiation to examine the chemical compositions and chemical valence states of the samples. N_2_ adsorption-desorption isotherms were obtained by an ASAP 2020 instrument at 77 K. The BET and QSDFT methods were respectively used to determine the specific surface areas and the pore size distributions of the materials.

### Electrochemical Measurements

The Co-ClNWs(NiE) electrode was treated under a pressure of 8 MPa, with the geometric area, mass load, and thickness of 1 cm^−2^, 3 mg, and 0.25 mm. respectively. To characterize the electrochemical behaviors of the (Co-ClNWs(NiE)), CHI660E (Chenhua, Shanghai) electrochemical workstation was used in a three-electrode electrochemical cell with a Pt counter electrode and a Hg/HgO reference electrode in 6 M KOH solution. Cyclic voltammetry (CV), galvanostatic charge and discharge (GCD) measurements were observed in the process of test. Electrochemical impedance spectroscopy (EIS) was tested by applying an AC voltage with 5 mV amplitude in a frequency range from 0.01 Hz to 100 kHz at open circuit potential. The products obtained in the same experiment but without growing on nickel foam were also collected for obtaining the working electrode plates (Co-ClNWs(E)) made by polytetrafluoroethylene (PTFE) sticking method. The specific capacitance of the samples was calculated according to Eq.(1):

1 $$ C=\frac{I\times \Delta t}{\Delta V\times m} $$

where *C* is the specific capacitance (F/g), *I* is the current (A), Δ*t* is the discharge time (s), Δ*V* is the potential window (V), and *m* is the mass of the electroactive electrode (g).

Moreover, an asymmetric supercapacitor with the Co-ClNWs(NiE) electrode (positive electrode) and activated carbon (AC, negative electrode) were tested in a two-electrode configuration. The optimal mass ratio for positive electrode to negative electrode was calculated by the equation below:

2 $$ {\mathrm{m}}_{+}/{\mathrm{m}}_{-}={\mathrm{C}}_{-}{\mathrm{V}}_{-}/{\mathrm{C}}_{+}{\mathrm{V}}_{+} $$

where *m* means the mass of active materials, *C* represents the gravimetric specific capacitance, and *V* is the potential window (in the three electrode configuration). To obtain the electrochemical performances, the specific capacitance, specific energy density, and specific power density of the cell were respectively calculated according to:

3 $$ {C}_c=\frac{I\varDelta t}{m\varDelta U} $$

4 $$ {E}_c=\frac{C_c\varDelta {U}^2}{2\times 3.6} $$

5 $$ {P}_c=\frac{E_c\times 3600}{\varDelta t} $$

where *I* (A) shows the charge/discharge current, *m* (g) represents the total active mass of the two electrodes, *Δt* (s) means the discharge time, *ΔU* (V) is the potential window, and *C*_*c*_ (F g^−1^), *E*_*c*_ (W h kg^−1^), and *P*_*c*_ (W kg^−1^) are the specific capacitance, energy density, and power density of the cell, respectively.

## Results and Discussion

### Characterization of the Co-ClNWs(NiE)

**Fig. 1:**
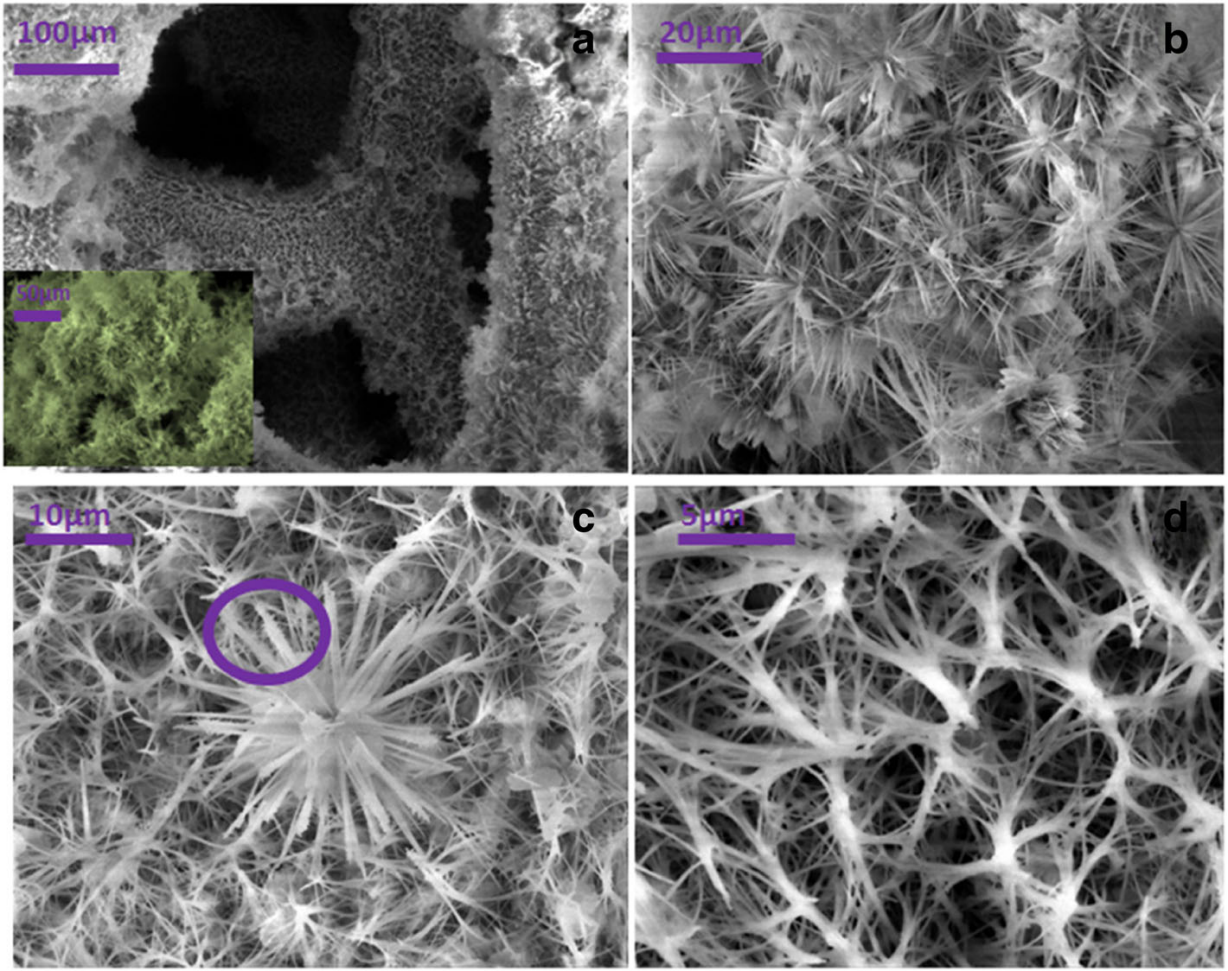
**a** Layered structure of nickel foam (the illustration shows the material attached to the nickel foam). **b** The appearance of the material observed under high magnification. **c** The monomeric flower morphology. **d** Material skeleton formed on the surface of foamed nickel

The SEM images in Fig. 1 show the morphology of the as-prepared Co-ClNWs on the nickel foam. In Fig. 1a, we can clearly know that nickel foam net itself has multiple layered structure. The electrode ligament formed by the three-dimensional structure of the nickel foam is very similar to that of the sponge, providing a natural frame work for the growth of materials [26]. The illustration shows the material is tightly covered on the nickel foam. The magnification of the image is presented in Fig. 1b, from which we found that the needle-shaped materials are staggered, demonstrating that the growth structure does not cause compression of the space structure, but forms a natural three-dimensional space gap. This distinctive structure can provide more pathways for the inflow and reaction of the electrolyte, which is beneficial for the electrode material in contact well with the electrolyte [27]. In Fig. 1c, observing the material grown on the surface of nickel foam, we find that the materials like the flourishing flowers are interconnected with each other, which is conductive to fast electron transport, thus improving the rate performance and reducing the energy loss. The magnification in Fig. 1d shows the surface of the nickel foam with the material skeleton by hydrothermal formation, and they exhibit an intertwined structure of orderly connection, which constitutes a tight knit conductive network. As we known, the electrode obtained by the PTFE sticking methods tends to cause problems such as uneven coating and does not have the natural spatial structure, which is easy to result in a drastic reduction of available space and specific surface area, reduces the utilization of experimental materials, and leads to significant performance differences ultimately [28]. Relative to that electrode, therefore, there is no doubt that the structure of Co-ClNWs(NiE) has the advantage of shortening the transmission distance of electrons and ions, so that the conductivity of the material is greatly improved, providing a good bedding for electrochemical test [29].

**Fig. 2:**
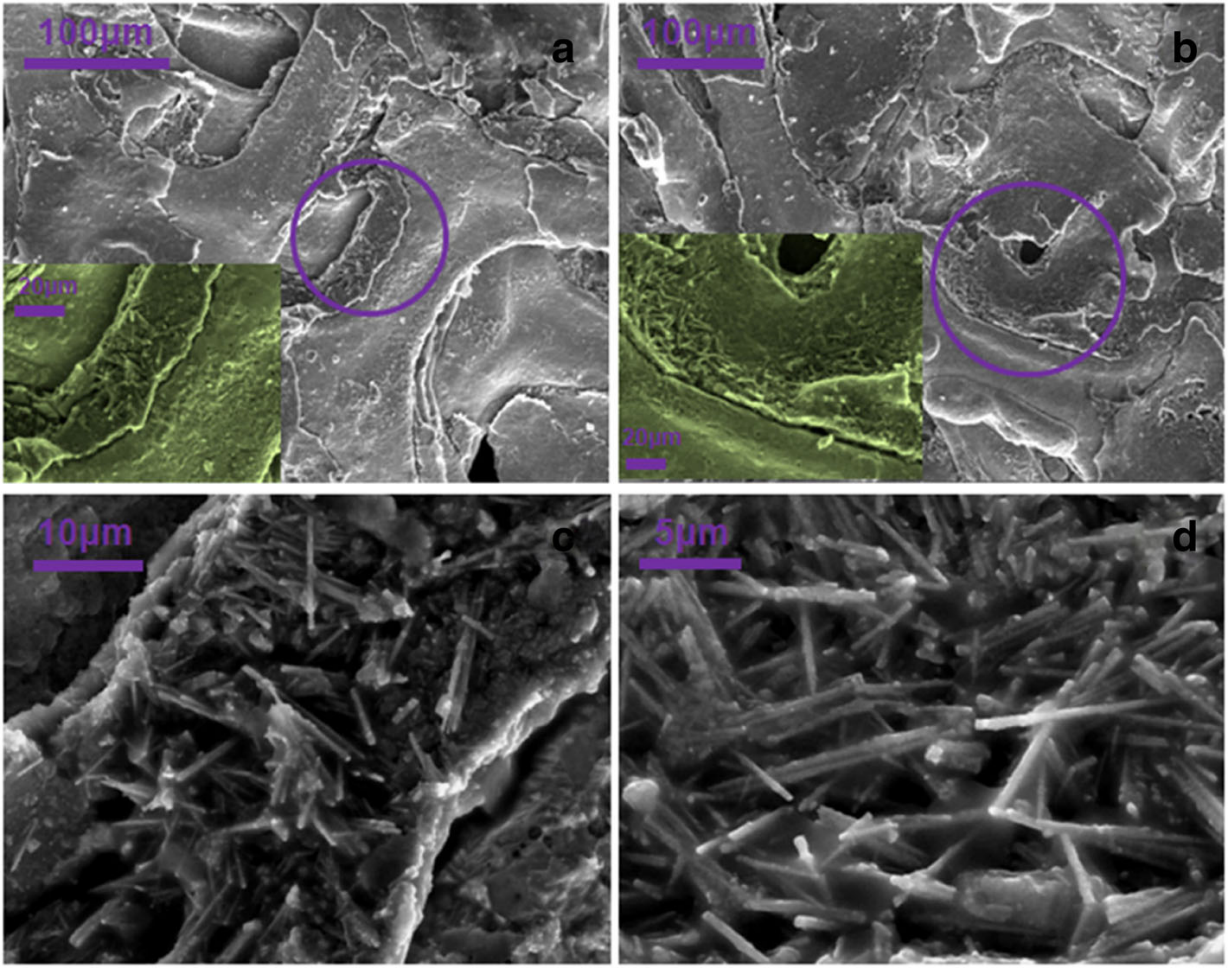
**a** SEM image of the tested Co-ClNWs(NiE) electrode. **b** SEM image of the material embedded in the nickel foam inter layers. **c**, **d** SEM images of closely aligned growth on the nickel foam skeleton under high magnification

To further explore the superiority of the Co-ClNWs(NiE) electrode, a SEM test is performed after the electrochemical test is completed. As seen from Fig. 2a, the nickel foam after the extrusion treatment is still hierarchical and the surface of the nickel foam is closely covered by the material. As we know, micro/nanometer scale protrusions are fabricated on commercial nickel foam (current collector), which can enhance its active sites [30]. A higher surface area of the current collector means more contact area between the current collector and the active material, which can boost the transportation of electrons and ions during electrochemical reactions. Good conductivity can ensure excellent rate capability for the capacitance under high current densities, so that the poor conductivity of the Co-based compound material is largely enhanced, confirmed by that many more needle-shaped nanowires are embedded in the interstices of nickel foam at high magnification [31]. In Fig. 2c, d, the enlarged image shows that the nanowires are arranged closely on the framework formed on the nickel foam so that the substrate space is fully utilized to exploit the active materials for storage energy. This is a structural advantage that is not possessed by Co-ClNWs(E) prepared by the PTFE sticking method. The Co-ClNWs(NiE) electrode preparation method offers a useful and viable approach which can fully stimulate the performance of the material.

**Fig. 3:**
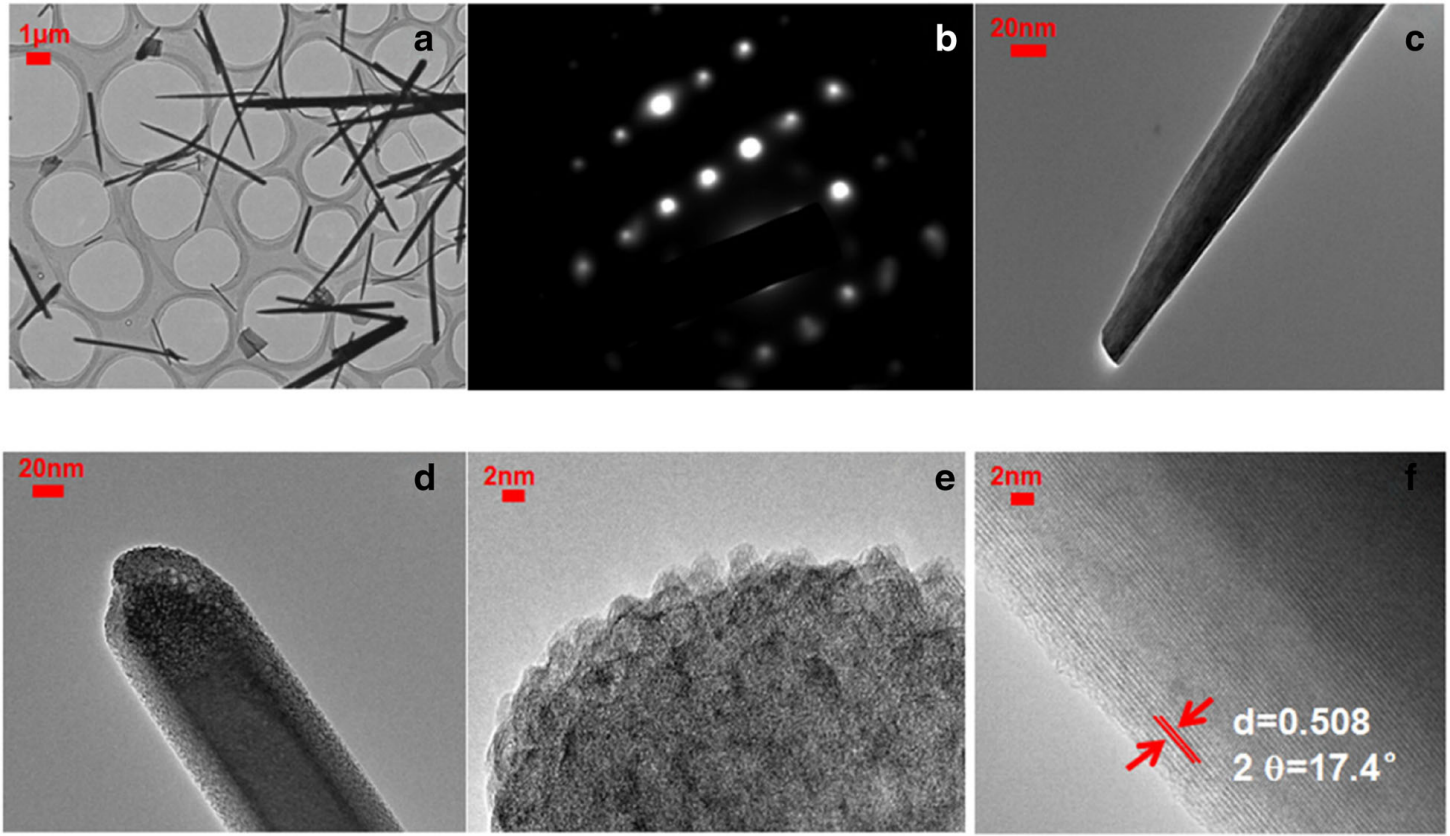
**a** TEM images of Co-ClNWs. **b** SEAD pattern of Co-ClNWs. **c**–**e** TEM images of Co-ClNWs at high magnification (showing surface mesoporous structure in (**d**), the particles are compactly arranged to form Co-ClNWs in (**e**)). **f** HRTEM images of the Co-ClNWs

The TEM pictures of Co-ClNWs nanowires (scraped from nickel foam products) are shown in Fig. 3. The image in Fig. 3a shows that the extracted material remains well acicular, which belongs to a single crystal structure as revealed by the electron diffraction (SAED) pattern of the selected nanowires in Fig. 3b. The acicular nanowires shown in Fig. 3c grow about a few microns in length with the diameter of about tens of nanometers, indicating a large aspect ratio. From the high magnification appearance of Co-ClNWs in Fig. 3d and Fig. 3e***,*** it is found that the surface of the material is closely aligned with the monomer whose diameter is about 3–10 nm. The deep diffusion of ions in the crystalline materials have always been considered a complex issue as electrolyte ions cannot diffuse throughout the material if the thickness of crystalline material is more than 30 nm. In our case, thus, the Co-ClNWs structure is conducive to the diffusion of the electrolyte because the size of the material monomer is about 3–10 nm, which shortens the diffusion distance of the electrolyte and reduces the reaction path length and resistance [32]. This factor dominantly allows effective utilization of all the material in Faradaic redox reaction. In addition, these arrangements make the material display a noticeable mesoporous appearance, which can greatly increase the ingratiation of electrolyte into the materials since the electrolyte ions cannot enter the ultrafine pores with the pore diameters below 2 nm though this pores can correspond to a higher specific surface area. Seen clearly from Fig. 3d, the pore size of the material is greater than 2 nm, which belongs to the category of mesopores and therefore is conductive to the electrolyte transportation [7]. As can be observed in Fig. 3f, the spacing of the lattice fringes is calculated to be ca.0.508 nm, which corresponds to 17.4° indexed to the XRD peak below according to the standard card of Co(CO_3_)_0.35_Cl_0.20_(OH)_1.10_1.74H_2_O (JDPS38-0547).

**Fig. 4:**
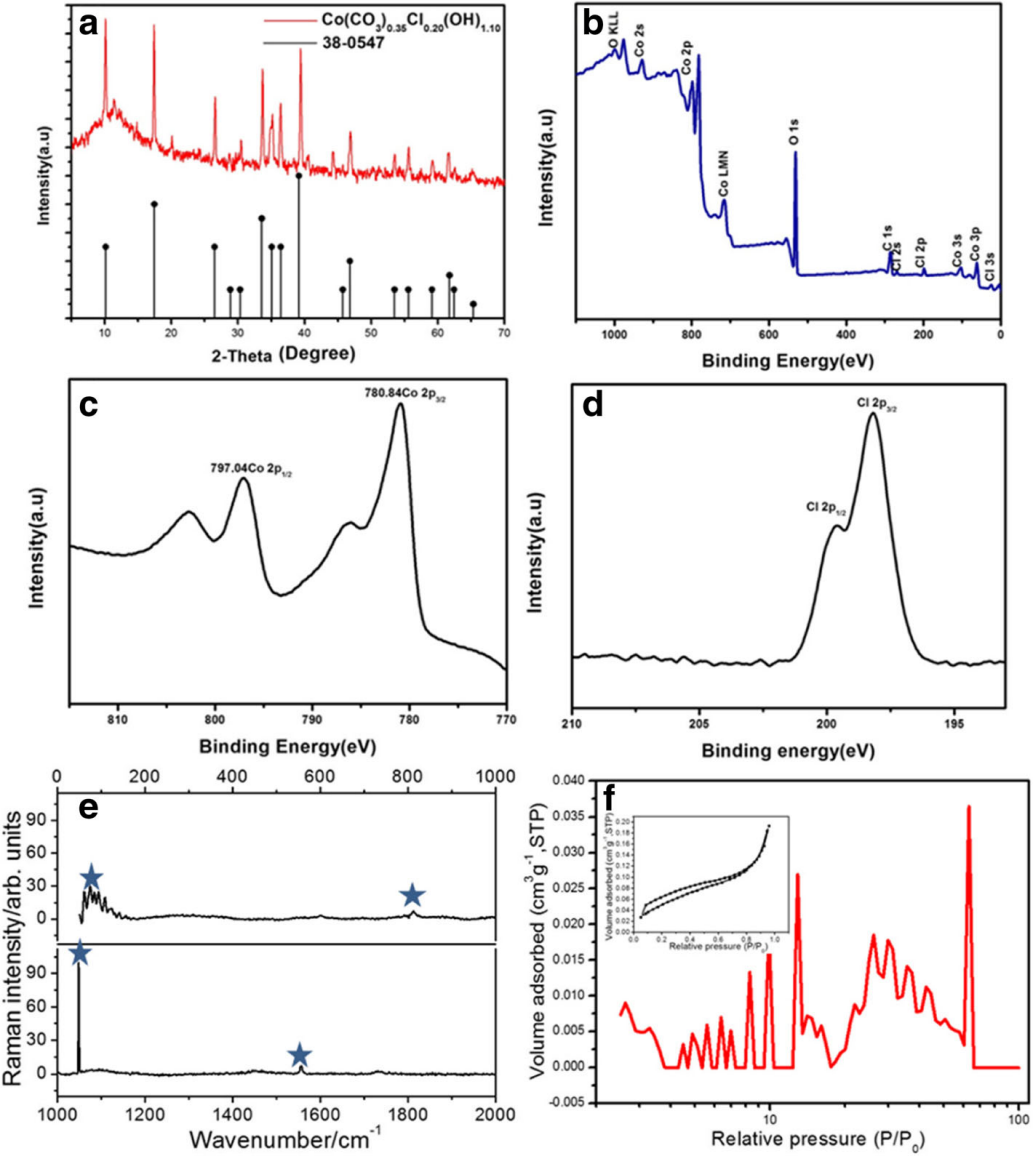
**a** XRD pattern, **b** XPS survey spectrum, **c** high resolution of Co 2p spectrum, **d** high resolution Cl 2p spectrum, **e** Raman spectrum, and **f** QSDFT pore size distribution (inset: N2 absorption/desorption isotherms) of Co-ClNWs

Figure 4a shows the XRD pattern of the material where all peaks are well-matched to the standard card (JCPDS 38-0547), confirming the nanowire stoichiometric composition is Co(CO_3_)_0.35_Cl_0.20_(OH)_1.10_. From the XPS scanning spectra in Fig. 4b, we find that the contents of Co, O, Cl, and C account for almost all the elements in the material, which demonstrates the high purity. The Co2p core X-ray photoelectron spectroscopy (XPS) spectrum (Fig. 4c) of the Co-ClNWs presents two major peaks at binding energies of 780.84 and 797.04 eV with a spin energy separation of ca. 16 eV. These two peaks correspond to Co2p_3/2_ and Co2p_1/2_ respectively and are accompanied by two obvious satellite peaks. The ionic state of the chlorine can also be inferred by the presence of spin-orbit doublets at 199.60 and 198.10 eV that can be identified as Cl2p_1/2_ and Cl2p_3/2_ signals, respectively (Fig. 4d).

In order to further obtain the constitution of the as-synthesized Co-ClNWs(NiE), Raman spectrum of it is illustrated in the wavenumber range from 0 to 2000 cm^−1^ and shown in Fig. 4e. Four Raman bands for Co-ClNWs(NiE) observed at about 95, 813, 1045, and 1554 cm^−1^ can be assigned to the bending mode for Cl-Co-Cl, Co-O-H deformation mode, -OH deformation mode, and ν_3_ (CO_3_)^2−^ antisymmetric stretching mode, respectively, suggesting that the main components are in agreement with the tests above [33–35]. The inset figure in Fig. 4f exhibits the N_2_ adsorption/desorption isotherm of Co-ClNWs(NiE), in which a type IV isotherm coupled with an obvious H3 character hysteresis loop can be observed, showing the existence of abundant meso- and macropores distribution onto the Co-ClNWs(NiE), in consistence with the result by TEM and the pore size distribution in Fig. 4f. This porous structure in terms of interconnected meso- and macropores is conductive to providing continuous channels for fast and unimpeded ion diffusion and thus ensuring a good accessibility of ion at the active sites. In addition, there are nearly no micropores existence in Co-ClNWs(NiE) because of nearly no N2 volume absorption under the pore sizes between 0 and 2 nm, which is responsible for the low specific surface area (about 5 m^2^/g) but for high crystallinity with rich active sites confirmed by XRD above.

### Electrochemical Performance of the Co-ClNWs(E) Electrode

**Fig. 5:**
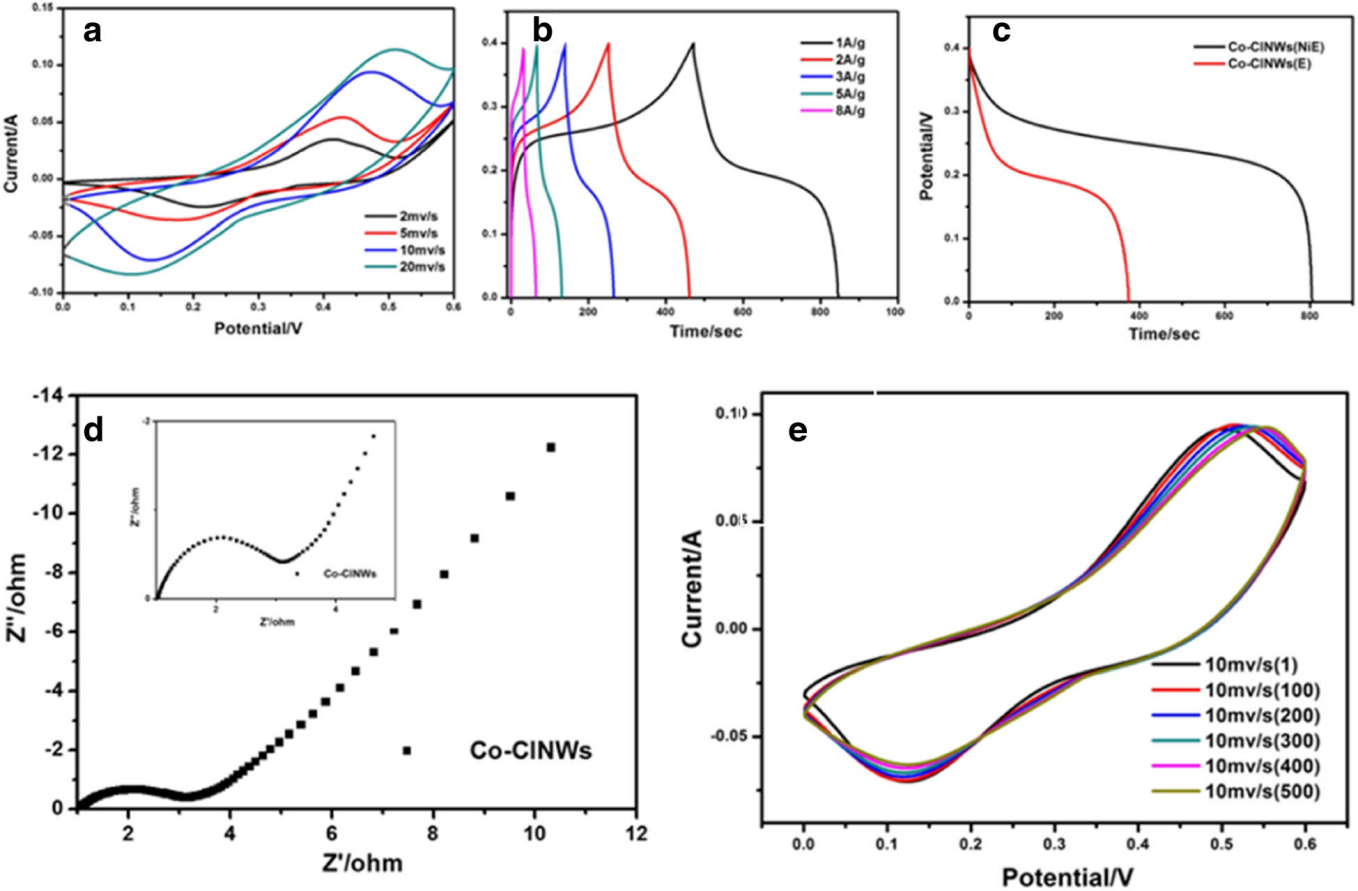
**a** CV curves of Co-ClNWs(E) at various scan rates. **b** Galvanostatic charge and discharge curves of Co-ClNWs(NiE) at various current densities. **c** Comparison of discharge curves of two electrodes. **d** The electrochemical impedance spectra of Co-ClNWs(E). **e** Comparison of CV curves after 500 cycles of Co-ClNWs(E)

The electrochemical behaviors of Co-ClNWs(E) are investigated by CV and GCD in a three electrode cell with a Hg/HgO reference electrode using 6 M KOH as aqueous electrolyte. Figure 5a corresponds to the CV curve obtained for Co-ClNWs(E) at sweep rates of 2, 5, 10, and 20 mv/s, in which all the CV curves are full and embody a symmetrical redox peak. With the increase of the scan rate, the peak position of the curve shifts, indicating that the capacitance performance comes from the activity material reactions, and nickel foam network is not involved in the relevant chemical reactions. The charge and discharge curves of Co-ClNWs(E) at different current densities are shown in Fig. 5b, with the typical characteristics agreeing well with the CV curves. The specific capacitance of the electrode material reaches 975, 950, 900, 825, and 640 F/g under the current density of 1, 2, 3, 5, and 8 A/g, respectively. Despite the better capacitive properties, there is a clear significant difference compared to Co-ClNWs (NiE), which is evident from Fig. 5c. Figure 5d shows the EIS spectrum of the Co-ClNWs(E) electrode, and it can be obtained that the Faradaic resistance reflected by the diameter of the semicircle is about 2 Ω. Such large resistance will inevitably lead to high hindrance of electron during the charge storage process. In Fig 5e, we perform a CV cycle test on Co-ClNWs(E) and find that the material is still able to exhibit a good and full redox curve, demonstrating the ability of the material to retain its properties after 500 test cycles. Hence, after investigating the electrochemical behavior of Co-ClNWs(E), we find that Co-ClNWs have the potential to become excellent capacitive material, and a better performances by promoting the application rate of active sites will be displayed if seeking a facile and effective way to improve the conductivity of it.

### Electrochemical Performance of the Co-ClNWs(NiE) Electrode

**Fig. 6:**
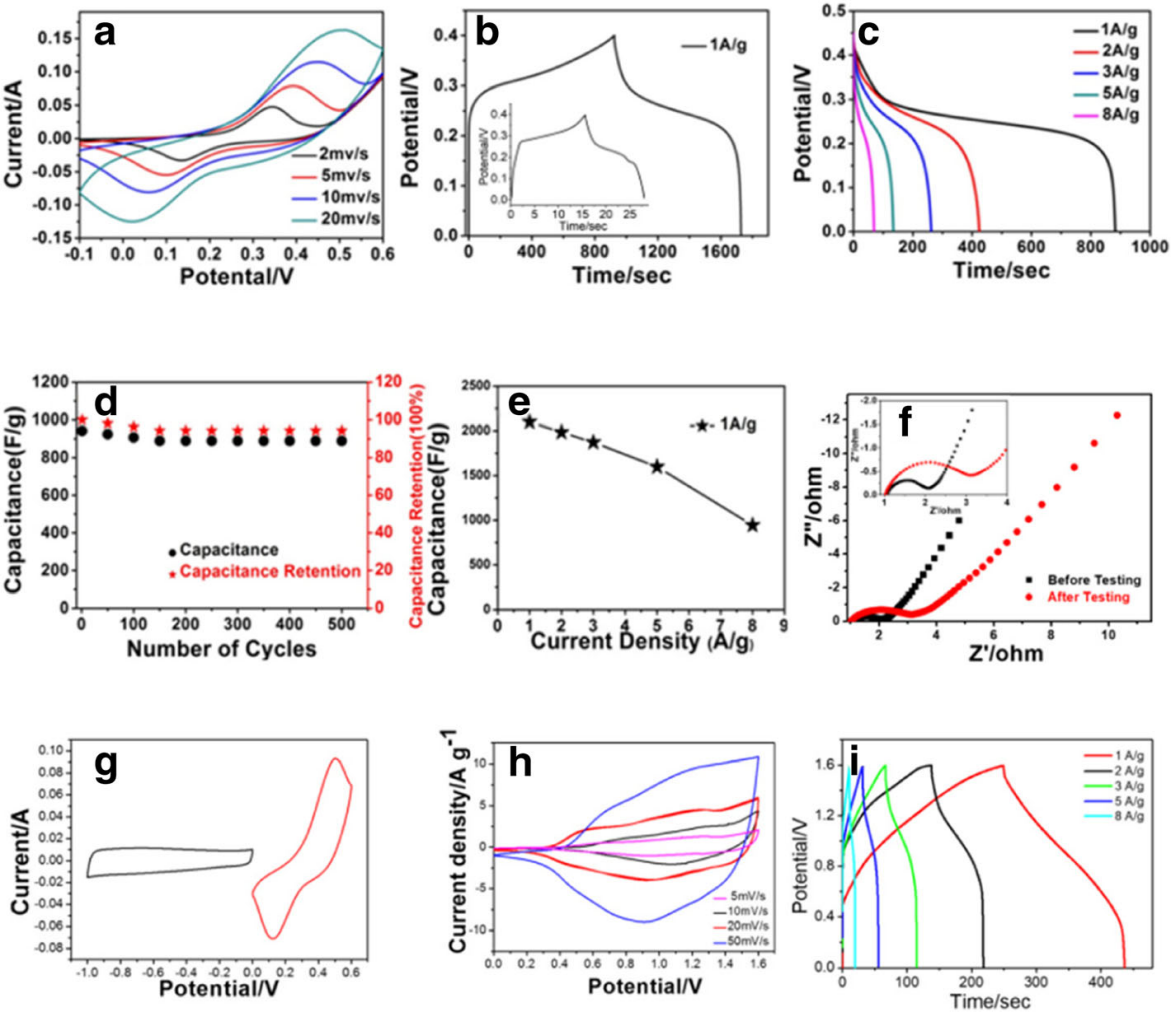
**a** CV curves of Co-Cl(NiE) at various scan rates. **b** Galvanostatic charge and discharge curve of Co-ClNWs(NiE) at 1 A/g (inset: GCD curve of nickel foam with the same current of Co-ClNWs(NiE) at 1A/g). **c** GCD curves of Co-ClNWs(NiE) at various current densities. **d** Long cyclic performance and capacitance retention of Co-ClNWs(NiE) at current densities of 8 A/g. **e** Average specific capacitance at various discharge current densities. **f** EIS spectra of Co-ClNWs(NiE) before and after electrochemical test over the frequency range from 100 kHz to 10 mHz. **g** CV curves of Co-ClNWs(NiE) and AC at a scan rate of 20 mV/s. **h** CV curves of the as-fabricated Co-ClNWs(NiE)//AC ASC device at various scan rates and the corresponding GCD curves (**i**) with different current densities

To examine the optimization of the Co-ClNWs(E) electrode, CV curves of Co-ClNWs(NiE) are tested with the same three-electrode configuration and shown in Fig. 6a*.* It can be observed that a full and neat curve is presented no matter what kind of scan rates from 2, 5, 10, and 20 mV/s. Moreover, each curve involves in a good redox symmetry, which fully demonstrates that the material has excellent pseudocapacitance characteristics [36]. As the scan rate increases, there is a reduced effective utilization area of the material with a slight shift in the peak, resulting in electrochemical performance decline due to the resistance and polarization of the electrode material [37, 38]. At higher scan rates, we can conclude that Co-ClNWs(NiE) has high rate capability because the redox peaks of the material species are still obvious. In addition, the current increases as the increasing of scan rates, which confirms the ability of it to conduct ions and electrons more efficiently. The main reason for the redox peaks is mainly attributed to the charge transfer between Co^2 +^/Co^3 +^ ions and the OH^−^ ions in the electrolyte involved in the reaction [39]. After reviewing the literature [40], the redox peaks correspond to the following reactions:

$$ {\displaystyle \begin{array}{l}\mathrm{Co}{\left(\mathrm{OH}\right)}_2\kern0.5em +{\mathrm{OH}}^{-}\leftrightarrow \mathrm{CoOOH}\kern0.5em +\kern0.5em {\mathrm{H}}_2\mathrm{O}+{e}^{-}\\ {}\mathrm{Co}\mathrm{OOH}\kern0.5em +\kern0.5em {\mathrm{OH}}^{-}\leftrightarrow {\mathrm{CoO}}_2\kern0.5em +{\mathrm{H}}_2\mathrm{O}\kern0.5em +\kern0.5em {e}^{-}\end{array}} $$

Figure 6b shows the GCD curve of the material at a current density of 1 A/g. It is found that the shape of the GCD curve has an obvious plateau, which proves that the material undergoes a redox reaction corresponding to the CV curves. It can be observed that the voltage drops suddenly due to the internal resistance of the material in the part of the discharge curve [41]. Moreover, we can also infer from Fig. 6b and Fig. 5c that the capacitances exhibited by the optimal sample Co-ClNWs(NiE) are higher than that by the addition of a single Co-ClNWs(E) and nickel foam, demonstrating that the combination of Co-ClNWs and nickel foam by direct growth have the promotion contributions to enhance the charge storage capability of the electrode, which means nickel foam can not only provide capacitance by itself but also can act as backbones to guarantee good electrical contact and mechanical adhesion and therefore increase the utilization rate of Co-ClNWs, as obviously seen in SEM figures in Fig. 1. Figure 6c gives the GCD curves at different current densities, with a specific capacitance of 2150 F/g at a current density of 1 A/g (higher than that of many recent published works in Table 1), corresponding to a ion specific capacitance of 4996 F/g of Co, which shows an excellent charge storage capability for Co-ClNWs(NiE) [42]. Additionally, the rate performance and the long-term stability of the electrode are further obtained according to the specific capacitances of the Co-ClNWs(NiE) electrode at different current densities and given in Fig. 6e. Although the performance of the capacitor decreases, the high power characteristic is still exhibited. The specific capacities of the capacitors are maintained at 1985, 1872, 1599, and 944 F/g at current densities of 2, 3, 5, and 8 A/g, respectively. The discharge capacitance is tested in Fig. 6d for multiple cycles to test the stability of Co-ClNWs(NiE), 94.3% of the specific capacitance at initial cycle of which can be maintained after 500 cycles. In our further test, however, the active material separation from the electrode is observed beyond 500 cycles, which may derived from the structure change of the bulk materials taking part in the Faradaic redox reaction, bringing about the inaccurate calculation of the specific capacitance based on the mass of Co-ClNWs under a given current density. In order to uncover such confused issue, therefore, our ongoing work will involve in tracking the reversibility of the electrochemical strains occurred during cycling. As shown in Fig. 6f, the electrochemical impedance spectra of the material before (MBT) and after (MAT) the test consist of a semicircle in the first half and a slash in the second half. It is generally accepted that the real axis intercept at high frequency represents the electrolyte resistance and the contact resistance between the active material and the current collector [43]. The straight line in the low-frequency area is ascribed to the ion diffusion resistance [30]. It can be seen that MBT has a smaller real axis intercept value at a high frequency than MAT, which means that MBT has a relatively smaller equivalent series resistance. In addition, it can be observed that the straight line for MBT has a higher slope than that for MAT, which also indicates that MBT can display a better ion diffusion. The slope of the two in the low-frequency region gradually tilts toward the *y*-axis, indicating that the electrolyte ions can rapidly diffuse into the pore structure of the material. The rate-controlling step of the reaction can be determined according to the electrochemical reaction on the surface of the electrode material such that the electrode material has good electrical properties.

**Table 1:** Comparison of the performance of our work with the literatures based on three-electrode system

Active materials	Electrolyte	Current density (A g^−1^)	Specific capacitance (F g^−1^)	Ref.
Ni_x_Co_3-x_O_4_	2 M KOH	1	1479	[47]
rGO/Co_2_(CO_3_)(OH)_2_)	3 M KOH	1	998	[48]
Cu_x_Co_2-x_CH	6 M KOH	1	789	[49]
Co_3_O_4_@MSDCN	6 M KOH	1	1307	[50]
Co_3_O_4_ nanowire	3 M KOH	0.5	418.5	[51]
GS/NiCo-LDH	6 M KOH	1	1980.7	[52]
NiCo-LDH/RGO	3 M KOH	2	1911.1	[53]
CNF@Ni-Co LDH	1 M NaOH	1	1378.2	[54]
Co-ClNWs(NiE)	6 M KOH	1	2150	This work

In order to further evaluate the charge storage capability of Co-ClNWs(NiE) in practice, an asymmetric supercapacitor (ASC) using Co-ClNWs(NiE) and AC respectively as positive electrode and negative electrode was fabricated. Figure 6g illustrates the CV curves of Co-ClNWs(NiE) and AC measured in a three-electrode system with the potential window of AC of − 1 to 0 V and Co-ClNWs(NiE) from 0 to 0.6 V. Therefore, it is expected that the as-fabricated ASC can be worked to 1.6 V. As shown in Fig. 6h, the CV curves of ASC under different scan rates show a pair of apparent peaks, demonstrating the typical faradaic characteristics [44]. Additionally, a specific capacitance of 117.5 F/g can be obtained from the GCD curve at 1 A/g in Fig. 6i, in accordance with a high energy density of 41.8 W h/kg at the power density of 1280.7 W/kg, higher than many recently publicized works [45, 46]. When the current density is enlarged to 8 A/g, the ACS can still exhibited an energy density of 21.2 W h/kg under a high power density of 6397.3 W/kg. This result clearly suggests that the ACS with the Co-ClNWs(NiE) as positive electrode exhibits a high energy density without sacrificing the high power density though a bulk redox reaction is involved, reflecting a possible method to keep a high energy storage capability under fast charge and discharge processes.

## Conclusion

In summary, a Co-ClNWs(NiE) electrode is fabricated via a facile one-step hydrothermal method. The active material Co-ClNWs is deposited on commercial nickel foam to form a free-standing supercapacitor electrode. After the optimization of the structure of the Co-ClNWs(E) electrode prepared by PTFE sticking method, the Co-ClNWs(NiE) electrode displays a high specific capacitance of 2150 F/g under the current density of 1 A/g, with a large energy density of 21.2 W h/kg under a high power density of 6397.3 W/kg even when the current density is up to 8 A/g. These results reveal that Co(CO_3_)_0.35_Cl_0__.20_(OH)_1.10_1.74H_2_O NWs are very promising candidates for the next generation of energy storage devices. On this basis, the structural advantages of nickel foam make the active materials fully reflect the capacitive properties. The electrode design concept described in this paper makes it possible to develop high-energy supercapacitors.

## Abbreviations

ASC: Asymmetric supercapacitor
Co-ClNWs: Chlorine-doped carbonated cobalt hydroxide nanowires
Co-ClNWs(E): Co-ClNWs stuck on the nickel foam
Co-ClNWs(NiE): Co-ClNWs grown on the nickel foam
CV: Cyclic voltammetry
EDLCs: Electrical double-layered capacitors
GCD: Galvanostatic charge and discharge measurements
MBT and MAT: The electrochemical impedance spectra of Co-ClNWs(NiE) before and after cycling
